# Mild Cognitive Impairment Subtypes Are Associated With Peculiar Gait Patterns in Parkinson’s Disease

**DOI:** 10.3389/fnagi.2022.781480

**Published:** 2022-03-01

**Authors:** Marianna Amboni, Carlo Ricciardi, Sofia Cuoco, Leandro Donisi, Antonio Volzone, Gianluca Ricciardelli, Maria Teresa Pellecchia, Gabriella Santangelo, Mario Cesarelli, Paolo Barone

**Affiliations:** ^1^Department of Medicine, Surgery and Dentistry, Center for Neurodegenerative Diseases (CEMAND), University of Salerno, Fisciano, Italy; ^2^Istituto di Diagnosi e Cura (IDC) Hermitage-Capodimonte, Naples, Italy; ^3^Department of Electrical Engineering and Information Technology, University of Naples Federico II, Naples, Italy; ^4^Istituti Clinici Scientifici Maugeri Istituto di Ricovero e Cura a Carattere Scientifico (IRCCS), Telese Terme, Italy; ^5^Department of Advanced Biomedical Sciences, University of Naples Federico II, Naples, Italy; ^6^Department of Medicine, Azienda Ospedaliera Universitaria OO. RR. San Giovanni di Dio e Ruggi D’Aragona, Salerno, Italy; ^7^Department of Psychology, University of Campania Luigi Vanvitelli, Caserta, Italy

**Keywords:** MCI (mild cognitive impairment), Parkinsion’s disease (PD), gait analysis, gait pattern characteristics, cognitive decline

## Abstract

**Background:**

Mild cognitive impairment (MCI) is frequent in Parkinson’s disease (PD) and represents a risk factor for the development of dementia associated with PD (PDD). Since PDD has been associated with disability, caregiver burden, and an increase in health-related costs, early detection of MCI associated with PD (PD-MCI) and its biomarkers is crucial.

**Objective:**

Given that gait is considered a surrogate marker for cognitive decline in PD, the aim of this study was to compare gait patterns in PD-MCI subtypes in order to verify the existence of an association between specific gait features and particular MCI subtypes.

**Methods:**

A total of 67 patients with PD were consecutively enrolled and assessed by an extensive clinical and cognitive examination. Based on the neuropsychological examination, patients were diagnosed as patients with MCI (PD-MCI) and without MCI (no-PD-MCI) and categorized in MCI subtypes. All patients were evaluated using a motion capture system of a BTS Bioengineering equipped with six IR digital cameras. Gait of the patients was assessed in the ON-state under three different tasks (a single task and two dual tasks). Statistical analysis included the *t*-test, the Kruskal–Wallis test with *post hoc* analysis, and the exploratory correlation analysis.

**Results:**

Gait pattern was poorer in PD-MCI vs. no-PD-MCI in all tasks. Among PD-MCI subtypes, multiple-domain PD-MCI and amnestic PD-MCI were coupled with worse gait patterns, notably in the dual task.

**Conclusion:**

Both the magnitude of cognitive impairment and the presence of memory dysfunction are associated with increased measures of dynamic unbalance, especially in dual-task conditions, likely mirroring the progressive involvement of posterior cortical networks.

## Introduction

Cognitive decline is frequent in Parkinson’s disease (PD) even in the early stages, and it occurs as a dysfunction in executive, attention, memory, language, and visuospatial domains (Barone et al., 2011). The full spectrum of cognitive skills in PD spans from normal cognition, through mild cognitive impairment (PD-MCI), to PD dementia (PDD) (Aarsland et al., 2017). A recent meta-analysis has shown that PD-MCI has a pooled prevalence of 40%, which is associated with older age, lower education, longer disease duration, higher levodopa equivalent daily dose, more severe motor symptoms, postural instability/gait difficulty motor subtype, poorer quality of life, and higher levels of apathy and depression (Baiano et al., 2020). In addition, PD-MCI represents a risk factor for the development of PDD (Hoogland et al., 2017; Saredakis et al., 2019), with a higher rate of conversion to dementia in the amnestic PD-MCI subtype relative to the non-amnestic PD-MCI subtype (Chung et al., 2019). Since PDD has been associated with increased disability, caregiver burden, and risk for institutionalization with a consequent increase in health-related costs (Svenningsson et al., 2012), the early detection of MCI and its biomarkers is crucial for the identification of a PD subpopulation at a higher risk of worse disease progression (Mollenhauer et al., 2014).

Cognition and gait in PD appear to be closely related in complex ways. Gait is no longer considered merely an automated motor task but an activity requiring multiple cognitive skills, ensuring safe mobility (Amboni et al., 2013). In a previous study, we have shown that dysfunctions in specific gait parameters that are poorly responsive to levodopa and are highly sensitive to dual-task conditions are associated with PD-MCI, and visuospatial impairment is strongly associated with instability in patients with PD (Amboni et al., 2012). On the one hand, these findings are in line with the hypothesis that dopa-resistant gait components and cognitive dysfunction might share common non-dopaminergic network dysfunction (Nonnekes et al., 2016); on the other hand, they are consistent with the recent findings that balance control would rely on posterior cortical networks (Morris et al., 2019). Moreover, we have provided insight into the chronological relationship between gait and cognitive decline in patients with PD, by showing that step length during a cognitive task on medication predicts subsequent executive/attention dysfunction (Amboni et al., 2018).

More recently, several studies have focused on the ability of gait analysis to distinguish subjects with MCI and different types of dementias. In particular, Mc Ardle et al. (2020) have shown that wearable sensors-based gait analysis was able to identify subjects with MCI, dementia with Lewy bodies (DLB), Alzheimer’s disease (AD), and PDD (Mc Ardle et al., 2020), whereas de Oliveira Silva et al. (2020) could distinct healthy controls and also patients with MCI and AD by using a videogrammetry system (de Oliveira Silva et al., 2020). In addition, gait-based machine learning approaches were able to discriminate different types of MCI, such as patients with PD-MCI and non-PD-MCI (Chen et al., 2020) or to detect the presence of MCI in PD (Ricciardi et al., 2020). Finally, Xie et al. (2019) employed an inertial-sensor-based wearable instrument to distinguish patients with amnestic MCI and cognitively normal subjects.

At present, studies comparing quantitative gait measures in PD-MCI subtypes are lacking. The main aim of this study was to compare gait patterns in PD-MCI subtypes in order to verify the existence of an association between specific gait features and particular MCI subtypes. This could shed further light both on the neurobiological substrate of different neuropsychological profiles and on the relationship between gait and cognition in PD.

## Materials and Methods

### Study Design and Population

The study sample consisted of 67 patients with PD, consecutively enrolled between February 2018 and June 2021. Participants were selected from patients referred to the Movement Disorders Unit of the Institute for Diagnosis and Care Hermitage-Capodimonte of Naples and the Center for Neurodegenerative Diseases of the University of Salerno. All patients fulfilled the Movement Disorder Society (MDS) clinical diagnostic criteria for PD (Postuma et al., 2015). The inclusion criteria were as follows: age ≥ 45 years; Hoehn and Yahr (H&Y) score ≤ 3; disease duration < 10 years; and antiparkinsonian treatment at a stable dosage during the previous 4 weeks. The exclusion criteria were as follows: gait requiring assistance; dementia according to the clinical diagnostic criteria for PDD (Emre et al., 2007); clinically significant comorbidities, including other neurological disorders, orthopedic diseases, or cardiovascular/respiratory diseases; anticholinergic or neuroleptic treatment; and brain surgery.

### Standard Protocol Approvals, Registrations, and Patient Consent

This study was performed in accordance with the 1964 Declaration of Helsinki and was approved by Campania Sud, the reference ethics committee of the Center for Neurodegenerative Diseases of the University of Salerno. Written informed consent was obtained from all subjects.

### Clinical and Cognitive Evaluations

All subjects were evaluated using a detailed assessment that included demographic, clinical, and anthropometric data. In addition, they completed the Italian version of the Montreal Cognitive Assessment (MoCA) and an extensive neuropsychological battery comprising the following tests: (1) the Rey Auditory 15-word Learning Test, immediate recall and delayed recall, and the Rey-Osterrieth complex figure delayed recall for the memory domain (Caltagirone et al., 1979; Caffarra et al., 2002); (2) the Stroop Color-Word Test and the Trail Making Test B-A for the attention domain (Giovagnoli et al., 1996; Barbarotto et al., 1998); (3) Phonological verbal fluency and the Clock Drawing Test for the executive domain (Caltagirone et al., 1979; Siciliano et al., 2016); (4) the Benton’s Judgment of Line Orientation and the Constructional apraxia test for the visuospatial domain (Spinnler and Tognoni, 1987; Ferracuti et al., 2000); and (5) Actions and objects naming tests for the language domain (Capasso and Miceli, 2001).

The test scores were corrected for current normative values. All neuropsychological tests were administered to the patients during the pharmacological ON-state. The diagnosis of PD-MCI by level II category and PD-MCI subtyping were based on the neuropsychological assessment according to the Movement Disorders Society Task Force guidelines (Litvan et al., 2012).

### Gait Analysis

All patients underwent gait analysis through a BTS Bioengineering system. The SMART DX is an optical system equipped with six IR cameras, two video cameras, two force plates, a set of passive markers, and an elaborator. The Davis protocol was used for all subjects, comprising the following four phases (Davis et al., 1991):

1. Collection of anthropometric measures of the patient (height, weight, and leg length).
2. Positioning of 22 reflective markers on specific points of the body of the patient.
3. The standing phase, consists of acquiring the patient while standing up on the force plate.
4. The walking phase on a straight path of 10 m during three different tasks including one single task and two dual tasks, namely:
  (a) GAIT: normal gait (single task).
  (b) MOT: walking while carrying a tray with two glasses filled with water (dual task).
  (c) COG: walking while serial subtracting 7s starting from 100 (dual task).

Each task was performed four times.

Prior to commencing the trials, all participants were trained to walk at a normal pace at their usual speed, without any instructions to prioritize walking or performing the concomitant task. This procedure generated a report from which spatial and temporal parameters were extracted.

All participants were evaluated in the self-defined best “ON-state” while receiving their typical dopaminergic drugs.

### Statistical Analysis

The statistical analysis was conducted by using Statistical Package for Social Science (SPSS, version 25). First, spatial and temporal variables of gait analysis were compared between patients with PD-MCI and with no-PD-MCI; then, they were compared among patients with no-PD-MCI, with single-domain PD-MCI, and with multiple-domain PD-MCI; and finally, they were compared among patients with no-PD-MCI, with non-amnestic PD-MCI, and with amnestic PD-MCI.

The *t*-test was used to compare two groups; since the analyzed samples contained more than 30 subjects, a normal distribution of the data is fair. Whereas, to compare three groups, given the small sample size for each group, the Kruskal–Wallis test was applied, and when significant, subsequent appropriate *post-hoc* tests were used in order to make the pairwise comparisons, thus correcting the *p*-values for multiple comparisons. Finally, an exploratory correlation study through the coefficient of Spearman was performed between spatiotemporal parameters and neuropsychological test scores.

Alpha significance level was set to *p* < 0.05 for all statistical analyses.

## Results

Thirty-two out of 67 patients were diagnosed as PD-MCI and 35 patients as no-PD-MCI. The 2 groups did not significantly differ on demographic and anthropometric variables, but they showed a trend toward significance on age (*p* = 0.053). When comparing clinical variables, the two groups differed significantly on the Movement Disorder Society Unified Parkinson’s Disease Rating Scale (MDS-UPDRS) part III (*p* = 0.014) and, consequently, showed a trend toward significance on total MDS-UPDRS (*p* = 0.050) (Table 1).

**TABLE 1:** Comparison of demographic and clinical features between patients with Parkinson’s disease (PD) without mild cognitive impairment (MCI) (no-PD-MCI) and with MCI (PD-MCI).

Variables	No-PD-MCI (*N* = 35)	PD-MCI (*N* = 32)	*p*-value
Age (years)	61.70 ± 7.36	65.70 ± 9.11	0.053
BMI	27.30 ± 2.95	28.2 ± 3.97	0.286
Disease duration (years)	4.46 ± 2.52	5.25 ± 2.50	0.207
Hoehn and Yahr	1.75 ± 0.41	1.92 ± 0.31	0.068
LEDD (mg)	495.00 ± 381.30	563.50 ± 380.20	0.465
MDS-UPDRS-Part I	6.54 ± 4.07	9.03 ± 7.07	0.088
MDS-UPDRS-Part II	6.97 ± 4.68	7.72 ± 5.64	0.556
MDS-UPDRS-Part III	20.1 ± 6.66	24.80 ± 8.65	**0.014**
MDS-UPDRS-Part IV	1.86 ± 3.02	1.44 ± 2.86	0.420
Total MDS-UPDRS	35.50 ± 12.10	43.00 ± 18.50	0.050

*PD, Parkinson’s Disease; MCI, Mild Cognitive Impairment; BMI, Body Mass Index; LEDD, Levodopa Equivalent Daily Dose; MDS-UPDRS, Movement Disorder Society Unified Parkinson’s Disease Rating Scale. Significant p-values are provided in bold.*

### Parkinson’s Disease-Mild Cognitive Impairment vs. No-Parkinson’s Disease-Mild Cognitive Impairment

Statistical *analyses* comparing all spatial and temporal gait parameters between patients with no-PD-MCI and PD-MCI are presented in Table 2.

**TABLE 2:** Comparison of all gait parameters (mean ± *SD*) between patients with PD without MCI (no-PD-MCI) and with MCI (PD-MCI).

Features	Unit of measure	No-PD-MCI	PD-MCI	*p*-value
**GAIT**				
Cycle duration	s	1.10 ± 0.11	1.09 ± 0.10	0.800
Stance duration	s	0.66 ± 0.07	0.67 ± 0.08	0.849
Swing duration	s	0.44 ± 0.05	0.43 ± 0.04	0.303
Swing duration variability	s	0.05 ± 0.06	0.04 ± 0.02	0.377
Stance phase	%	59.90 ± 2.24	60.90 ± 1.64	0.054
Swing phase	%	39.60 ± 1.69	39.20 ± 1.62	0.279
Single-support phase	%	39.40 ± 2.60	39.20 ± 1.63	0.639
Double-support phase	%	10.20 ± 1.57	11.30 ± 3.29	0.074
Mean velocity	m/s	1.07 ± 0.15	0.99 ± 0.16	0.075
Mean velocity	% height/s	63.30 ± 9.01	60.20 ± 10.00	0.179
Cadence	steps/min	108.60 ± 12.60	110.80 ± 10.50	0.442
Cycle length	m	1.160 ± 0.12	1.07 ± 0.15	**0.007**
Cycle length	% height	69.70 ± 6.08	65.2 ± 10.13	**0.031**
Step length	m	0.56 ± 0.09	0.48 ± 0.13	**0.007**
Step length variability	m	0.17 ± 0.39	0.33 ± 0.54	0.169
Step width	m	0.09 ± 0.04	0.11 ± 0.07	0.312
**MOT**				
Cycle duration	s	1.09 ± 0.12	1.07 ± 0.10	0.403
Stance duration	s	0.66 ± 0.08	0.66 ± 0.07	0.903
Swing duration	s	0.44 ± 0.50	0.45 ± 0.19	0.677
Swing duration variability	s	0.04 ± 0.03	0.03 ± 0.02	0.715
Stance phase	%	60.14 ± 1.45	61.12 ± 2.20	**0.039**
Swing phase	%	39.86 ± 1.45	38.86 ± 2.20	**0.035**
Single-support phase	%	39.93 ± 1.59	38.85 ± 2.17	**0.026**
Double-support phase	%	11.37 ± 3.24	11.96 ± 2.81	0.431
Mean velocity	m/s	1.05 ± 0.15	0.98 ± 0.19	0.077
Mean velocity	% height/s	62.64 ± 8.88	59.22 ± 10.49	0.154
Cadence	steps/min	111.10 ± 12.32	112.80 ± 10.16	0.528
Cycle length	m	1.13 ± 0.13	1.03 ± 0.17	**0.013**
Cycle length	% height	67.73 ± 6.97	63.07 ± 10.45	**0.034**
Step length	m	0.55 ± 0.08	0.48 ± 0.13	**0.009**
Step length variability	m	0.10 ± 0.18	0.25 ± 0.57	0.163
Step width	m	0.10 ± 0.05	0.11 ± 0.06	0.850
**COG**				
Cycle duration	S	1.14 ± 0.14	1.17 ± 0.16	0.358
Stance duration	s	0.70 ± 0.09	0.74 ± 0.11	0.146
Swing duration	s	0.44 ± 0.05	0.44 ± 0.06	0.902
Swing duration variability	s	0.03 ± 0.02	0.06 ± 0.05	**0.022**
Stance phase	%	61.30 ± 1.89	62.70 ± 2.19	**0.009**
Swing phase	%	38.70 ± 1.90	38.60 ± 5.27	0.898
Single-support phase	%	38.30 ± 2.83	37.70 ± 2.21	0.339
Double-support phase	%	11.70 ± 1.66	13.90 ± 4.10	**0.007**
Mean velocity	m/s	0.95 ± 0.16	0.81 ± 0.18	**0.001**
Mean velocity	% height/s	57.30 ± 9.33	49.50 ± 11.20	**0.003**
Cadence	steps/min	106.60 ± 12.90	104.10 ± 13.50	0.450
Cycle length	m	1.07 ± 0.12	0.94 ± 0.18	**0.001**
Cycle length	% height	64.50 ± 6.47	57.50 ± 12.90	**0.006**
Step length	m	0.53 ± 0.08	0.41 ± 0.12	**0.000**
Step length variability	m	0.09 ± 0.24	0.37 ± 0.50	**0.006**
Step width	m	0.12 ± 0.12	0.12 ± 0.07	0.808

*PD, Parkinson’s Disease; MCI, Mild Cognitive Impairment; GAIT, normal gait; MOT, walking while carrying a tray with two glasses filled of water; COG, walking while serial subtracting 7s starting from 100. Significant p-values are provided in bold.*

In all three tasks, patients with PD-MCI exhibited poorer gait patterns when compared with patients with no-PD-MCI. In comparison with patients with no-PD-MCI, patients with PD-MCI exhibited shortened cycle and step length in all tasks (*p* < 0.05). In addition, in the MOT task, PD-MCI vs. no-PD-MCI showed increased stance phase and reduced swing and single-support phases (*p* < 0.05), whereas, in the COG task, they exhibited increased stance phase mainly due to longer double-support stance phase duration, increased swing and step variabilities, and reduced velocity (*p* > 0.05).

### No-Parkinson’s Disease-Mild Cognitive Impairment vs. Single-Domain Parkinson’s Disease-Mild Cognitive Impairment vs. Multiple-Domain Parkinson’s Disease-Mild Cognitive Impairment

When subtyping PD-MCI according to the number of affected cognitive domains, 7 patients were categorized as single-domain PD-MCI and 25 patients as multiple-domain PD-MCI. Table 3 shows the comparison of all spatial and temporal gait parameters among no-PD-MCI, single-domain PD-MCI, and multiple-domain PD-MCI with subsequent *post-hoc* tests when appropriate.

**TABLE 3:** Comparison of all gait parameters (mean ± *SD*) among no-PD-MCI, single-domain PD-MCI, and multiple-domain PD-MCI.

Features	No-PD-MCI (0)	Single-domain PD-MCI (1)	Multiple-domain PD-MCI (2)	*p*-value Kruskal–wallis	*p*-value *post hoc*
**GAIT**					
Cycle duration	1.10 ± 0.11	1.09 ± 0.10	1.09 ± 0.10	0.986	
Stance duration	0.66 ± 0.07	0.66 ± 0.06	0.67 ± 0.08	0.998	
Swing duration	0.44 ± 0.05	0.44 ± 0.04	0.43 ± 0.03	0.692	
Swing duration variability	0.05 ± 0.06	0.05 ± 0.01	0.03 ± 0.02	0.101	
Stance phase	59.90 ± 2.24	60.10 ± 1.07	61.00 ± 1.70	0.159	
Swing phase	39.60 ± 1.69	39.90 ± 1.05	39.00 ± 1.70	0.337	
Single-support phase	39.40 ± 2.60	39.90 ± 1.05	39.00 ± 1.70	0.311	
Double-support phase	10.20 ± 1.57	10.20 ± 1.47	11.5 ± 3.56	0.279	
Mean velocity	1.07 ± 0.15	1.08 ± 0.08	0.98 ± 0.17	0.142	
Mean velocity% h/s	63.30 ± 9.01	64.00 ± 5.05	59.30 ± 10.7	0.266	
Cadence	108.60 ± 12.60	110.80 ± 9.67	110.80 ± 10.90	0.829	
Cycle length	1.16 ± 0.12	1.18 ± 0.04	1.05 ± 0.15	**0.020**	**2-0 0.032**
Cycle length % h	69.70 ± 6.08	69.30 ± 1.73	64.20 ± 11.00	**0.005**	**2-0 0.005**
Step length	0.56 ± 0.09	0.57 ± 0.07	0.46 ± 0.13	**0.012**	**2-0 0.015**
Step length variability	0.17 ± 0.39	0.13 ± 0.22	0.38 ± 0.59	0.263	
Step width	0.09 ± 0.04	0.10 ± 0.05	0.11 ± 0.07	0.933	
**MOT**					
Cycle duration	1.09 ± 0.12	1.05 ± 0.09	1.07 ± 0.10	0.632	
Stance duration	0.66 ± 0.08	0.63 ± 0.06	0.66 ± 0.07	0.478	
Swing duration	0.44 ± 0.50	0.42 ± 0.04	0.46 ± 0.21	0.391	
Swing duration variability	0.04 ± 0.03	0.03 ± 0.03	0.03 ± 0.02	0.381	
Stance phase	60.10 ± 1.45	59.50 ± 2.17	61.50 ± 2.07	**0.024**	1-2 0.096 0-2 0.064
Swing phase	39.90 ± 1.45	40.50 ± 2.17	38.50 ± 2.07	**0.019**	**2-0 0.050**
Single-support phase	39.90 ± 1.59	40.50 ± 2.19	38.50 ± 2.02	**0.015**	**2-0 0.039**
Double-support phase	11.40 ± 3.24	11.40 ± 3.39	12.10 ± 2.72	0.210	
Mean velocity	1.05 ± 0.15	1.12 ± 0.17	0.94 ± 0.19	**0.034**	2-0 0.088 2-1 0.119
Mean velocity % h/s	62.60 ± 8.90	66.50 ± 9.50	57.60 ± 10.15	**0.034**	2-0 0.105 2-1 0.101
Cadence	111.10 ± 12.30	115.33 ± 9.90	112.30 ± 10.30	0.665	
Cycle length	1.13 ± 0.13	1.16 ± 0.08	1.00 ± 0.17	**0.008**	**2-0 0.015**
Cycle length % h	67.70 ± 6.90	68.80 ± 5.70	61.70 ± 10.90	**0.003**	**2-0 0.003**
Step length	0.55 ± 0.08	0.56 ± 0.06	0.46 ± 0.13	**0.018**	**2-0 0.015**
Step length variability	0.10 ± 0.18	0.13 ± 0.22	0.28 ± 0.62	0.659	
Step width	0.10 ± 0.05	0.11 ± 0.06	0.11 ± 0.06	0.815	
**COG**					
Cycle duration	1.14 ± 0.14	1.18 ± 0.16	1.18 ± 0.16	0.677	
Stance duration	0.70 ± 0.09	0.72 ± 0.08	0.74 ± 0.11	0.396	
Swing duration	0.44 ± 0.05	0.46 ± 0.09	0.43 ± 0.05	0.998	
Swing duration variability	0.03 ± 0.02	0.05 ± 0.04	0.06 ± 0.05	**0.024**	**2-0 0.022**
Stance phase	61.30 ± 1.89	61.60 ± 2.21	62.90 ± 2.15	**0.019**	**2-0 0.015**
Swing phase	38.70 ± 1.90	43.60 ± 10.70	37.40 ± 2.02	**0.016**	2-0 0.061 2-1 0.052
Single-support phase	38.30 ± 2.83	38.70 ± 2.41	37.50 ± 2.14	0.137	
Double-support phase	11.70 ± 1.66	13.90 ± 3.65	13.90 ± 4.26	**0.030**	**2-0 0.039**
Mean velocity	0.95 ± 0.16	0.92 ± 0.12	0.79 ± 0.19	**0.002**	**2-0 0.001**
Mean velocity % h/s	57.30 ± 9.33	53.80 ± 7.57	48.50 ± 11.80	**0.008**	**2-0 0.006**
Cadence	106.50 ± 12.90	104.20 ± 12.90	104.10 ± 13.90	0.732	
Cycle length	1.07 ± 0.12	1.05 ± 0.09	0.92 ± 0.19	**0.002**	**2-0 0.002**
Cycle length % h	64.50 ± 6.47	62.1 ± 7.28	56.40 ± 13.80	**0.001**	**2-0 0.001**
Step length	0.53 ± 0.08	0.44 ± 0.10	0.40 ± 0.12	**0.000**	**2-0 0.000**
Step length variability	0.09 ± 0.24	0.39 ± 0.39	0.37 ± 0.53	**0.002**	**2-0 0.004**
Step width	0.12 ± 0.12	0.14 ± 0.11	0.11 ± 0.07	0.796	

*No-PD-MCI, Patients with Parkinson’s Disease without Mild Cognitive Impairment; PD-MCI, Patients with Parkinson’s Disease with Mild Cognitive Impairment; GAIT, normal gait; MOT, walking while carrying a tray with two glasses filled of water; COG, walking while serial subtracting 7s starting from 100. Significant p-values are provided in bold.*

In all three tasks, step and cycle lengths showed statistically significant differences among the three groups (*p* < 0.05); *post hoc* comparisons revealed significantly shortened step and cycle lengths in multiple-domain PD-MCI vs. no-PD-MCI (*p* < 0.05). Moreover, in the MOT task, the stance phase, the swing phase, the single-support phase, and velocity resulted statistically different among the three groups; the *post hoc* analysis disclosed reduced swing and single-support phases in multiple-domain PD-MCI vs. no-PD-MCI (*p* < 0.05).

In the COG task, swing phase and step length variabilities, the stance and swing phases, the double-support phase, and velocity were significantly different among the three groups (*p* < 0.05); the *post hoc* analysis revealed increased swing phase and step length variabilities, longer stance and double-support phases, and reduced velocity in multiple-domain PD-MCI as compared to no-PD-MCI (*p* < 0.05).

### No-Parkinson’s Disease-Mild Cognitive Impairment vs. Amnestic Parkinson’s Disease-Mild Cognitive Impairment vs. Non-amnestic Parkinson’s Disease-Mild Cognitive Impairment

When subtyping PD-MCI based on the presence of memory impairment, 21 patients were diagnosed as amnestic PD-MCI and 11 patients as non-amnestic PD-MCI. Table 4 shows the comparison of all spatial and temporal gait parameters among no-PD-MCI, amnestic PD-MCI, and non-amnestic PD-MCI using subsequent *post-hoc* tests when appropriate.

**TABLE 4:** Comparison of all gait parameters (mean ± *SD*) among no-PD-MCI, amnestic PD-MCI, and non-amnestic PD-MCI.

Features	No-PD-MCI (0)	Amnestic PD-MCI (1)	Non-amnestic PD-MCI (2)	*p*-value Kruskal–wallis	*p*-value *post hoc*
**GAIT**					
Cycle duration	1.10 ± 0.11	1.11 ± 0.10	1.05 ± 0.10	0.272	
Stance duration	0.66 ± 0.07	0.68 ± 0.08	0.64 ± 0.06	0.235	
Swing duration	0.44 ± 0.05	0.43 ± 0.03	0.42 ± 0.05	0.337	
Swing duration variability	0.05 ± 0.06	0.04 ± 0.02	0.03 ± 0.02	0.995	
Stance phase	59.90 ± 2.24	61.1 ± 1.75	60.40 ± 1.33	0.147	
Swing phase	39.60 ± 1.69	38.90 ± 1.75	39.60 ± 1.32	0.324	
Single-support phase	39.40 ± 2.60	38.90 ± 1.76	39.70 ± 1.31	0.304	
Double-support phase	10.20 ± 1.57	11.80 ± 3.83	10.20 ± 1.53	0.121	
Mean velocity	1.07 ± 0.15	0.97 ± 0.18	1.04 ± 0.10	0.333	
Mean velocity % h/s	63.3 ± 9.01	59.10 ± 11.70	62.20 ± 5.41	0.368	
Cadence	108.60 ± 12.60	108.50 ± 10.10	115.00 ± 10.60	0.241	
Cycle length	1.16 ± 0.12	1.07 ± 0.17	1.09 ± 0.12	0.125	
Cycle length % h	69.7 ± 6.08	65.10 ± 11.70	65.40 ± 6.68	**0.022**	**1-0 0.034**
Step length	0.56 ± 0.10	0.48 ± 0.13	0.47 ± 0.14	0.065	
Step length variability	0.17 ± 0.39	0.34 ± 0.61	0.32 ± 0.42	0.254	
Step width	0.09 ± 0.04	0.09 ± 0.05	0.14 ± 0.09	0.508	
**MOT**					
Cycle duration	1.09 ± 0.12	1.09 ± 0.09	1.03 ± 0.10	0.220	
Stance duration	0.66 ± 0.08	0.68 ± 0.08	0.62 ± 0.05	0.116	
Swing duration	0.44 ± 0.50	0.47 ± 0.23	0.41 ± 0.05	0.204	
Swing duration variability	0.04 ± 0.03	0.03 ± 0.02	0.03 ± 0.02	0.928	
Stance phase	60.10 ± 1.45	61.50 ± 2.31	60.50 ± 1.91	0.174	
Swing phase	39.90 ± 1.45	38.50 ± 2.30	39.50 ± 1.91	0.157	
Single-support phase	39.90 ± 1.59	28.50 ± 2.28	39.50 ± 1.88	0.143	
Double-support phase	11.40 ± 3.24	12.20 ± 3.06	11.60 ± 2.35	0.387	
Mean velocity	1.05 ± 0.15	0.95 ± 0.22	1.01 ± 0.12	0.245	
Mean velocity % h/s	62.60 ± 8.90	58.10 ± 10.30	61.3 ± 6.59	0.311	
Cadence	111.10 ± 12.30	110.40 ± 9.00	117.60 ± 10.90	0.198	
Cycle length	1.13 ± 0.13	1.03 ± 0.19	1.05 ± 0.12	0.086	
Cycle length % h	67.70 ± 6.97	63.20 ± 12.10	62.90 ± 6.96	**0.022**	1-0 0.076 2-0 0.076
Step length	0.55 ± 0.08	0.48 ± 0.13	0.48 ± 0.12	0.072	
Step length variability	0.10 ± 0.18	0.27 ± 0.66	0.22 ± 0.34	0.637	
Step width	0.10 ± 0.05	0.10 ± 0.06	0.11 ± 0.06	0.839	
**COG**					
Cycle duration	1.14 ± 0.14	1.21 ± 0.15	1.11 ± 0.16	0.108	
Stance duration	0.70 ± 0.09	0.77 ± 0.10	0.69 ± 0.10	**0.048**	1-0 0.095
Swing duration	0.44 ± 0.05	0.45 ± 0.05	0.43 ± 0.07	0.356	
Swing duration variability	0.03 ± 0.02	0.06 ± 0.06	0.04 ± 0.02	**0.021**	**1-0 0.021**
Stance phase	61.30 ± 1.90	63.20 ± 1.9	61.80 ± 2.40	**0.013**	**1-0 0.009**
Swing phase	38.70 ± 1.90	38.50 ± 6.40	38.60 ± 1.90	0.129	
Single-support phase	38.30 ± 2.80	37.30 ± 2.20	38.60 ± 1.90	0.095	
Double-support phase	11.70 ± 1.70	14.1 ± 4.20	13.70 ± 4.00	**0.024**	**1-0 0.025**
Mean velocity	0.95 ± 0.16	0.79 ± 0.19	0.84 ± 0.17	**0.005**	**1-0 0.006**
Mean velocity % h/s	57.30 ± 9.30	48.70 ± 11.70	51.00 ± 10.50	**0.011**	**1-0 0.011**
Cadence	106.50 ± 12.90	100.90 ± 12.10	110.00 ± 14.60	0.127	
Cycle length	1.07 ± 0.12	0.94 ± 0.17	0.94 ± 0.22	**0.009**	**1-0 0.015**
Cycle length % h	64.50 ± 6.50	57.90 ± 13.10	56.70 ± 13.40	**0.004**	**1-0 0.006**
Step length	0.53 ± 0.08	0.40 ± 0.13	0.42 ± 0.10	**0.000**	**2-0 0.009 1-0 0.001**
Step length variability	0.09 ± 0.24	0.44 ± 0.57	0.23 ± 0.32	**0.001**	**1-0 0.001**
Step width	0.11 ± 0.12	0.11 ± 0.07	0.12 ± 0.08	0.800	

*No-PD-MCI, Patients with Parkinson’s Disease without Mild Cognitive Impairment; PD-MCI, Patients with Parkinson’s Disease with Mild Cognitive Impairment; GAIT, normal gait; MOT, walking while carrying a tray with two glasses filled of water; COG, walking while serial subtracting 7s starting from 100. Significant p-values are provided in bold.*

In all three tasks, the cycle length normalized for height resulted to be significantly different among the three groups (*p* < 0.05); in the GAIT and COG tasks, *post hoc* comparisons revealed significantly shortened cycle length normalized for height in amnestic PD-MCI vs. no-PD-MCI (*p* < 0.05). In addition, in the COG task, swing phase and step length variabilities, stance duration, stance and double-support phases, velocity, and cycle and step lengths were significantly different among the three groups (*p* < 0.05). The *post hoc* analysis revealed increased swing phase and step length variabilities, increased stance and double-support phases, reduced velocity, and shortened cycle and step lengths in amnestic PD-MCI as compared to no-PD-MCI (*p* < 0.05); the *post hoc* analysis also unveiled shortened step length in non-amnestic PD-MCI vs. no-PD-MCI (*p* < 0.001).

### Exploratory Correlation Analysis Between Gait Parameters and Neuropsychological Test Scores

The correlation analysis was performed between the neuropsychological tests scores and the spatiotemporal gait parameters under the COG dual task, as these gait variables were most related to cognition in the previous analysis. Significant correlations were obtained for the global cognition scale MoCA, which correlated with the cycle duration (*r* = −0.664, *p* = 0.004), the stance duration (*r* = −0.678, *p* = 0.003), the stance phase (*r* = −0.540, *p* = 0.025), the swing phase (*r* = 0.554, *p* = 0.021), the single-support phase (*r* = 0.554, *p* = 0.021), the double-support phase (*r* = −0.537, *p* = 0.026), and the cadence (*r* = −0.660, *p* = 0.004).

Among the domain-specific neuropsychological tests, only the Benton’s Judgment of Line Orientation Test exhibited a significant correlation (between 0.500 and 0.600) with a gait parameter, namely mean velocity (*r* = 0.550, *p* < 0.001) (Supplementary Figure 1).

## Discussion

To our knowledge, this is the first study evaluating quantitative walking parameters in MCI subtypes in PD. After confirming that PD-MCI is associated with the dysfunction of several spatial and temporal gait parameters, especially in dual-task conditions, in this study, we showed that multiple-domain PD-MCI and amnestic PD-MCI are coupled with worse gait patterns.

### Comparison of Gait Features in Parkinson’s Disease-Mild Cognitive Impairment vs. No-Parkinson’s Disease-Mild Cognitive Impairment

Consistent with previous findings (Amboni et al., 2012, 2018; Morris et al., 2016, 2019), patients with PD-MCI as compared to patients with no-PD-MCI showed predominant dysfunctions on spatial gait features, i.e., reduced cycle and step length, which were evident in both single and dual task. Under dual-task conditions, PD-MCI exhibited increased stance phase, mainly in double support, and augmented the measures of variability, i.e., swing duration variability and step length variability, which are all indicators of dynamic instability (Plotnik et al., 2011). These findings further support the detrimental effect of the dual task on gait performance, especially on dynamic balance, in patients with cognitive decline (Amboni et al., 2013; Bishnoi and Hernandez, 2020; Ricciardi et al., 2020) with a subsequent increased risk of falling. In fact, the sensitizing role of dual task relies on its peculiar mechanism that leads to a competition for attention resources that collapse when a cognitive reserve is reduced (Amboni et al., 2013). It is worth noting that, since all patients were on medication during gait analysis, the observed gait variable dysfunctions may mirror their levodopa-resistant nature.

### Comparison of Gait Features in Parkinson’s Disease-Mild Cognitive Impairment Subtypes

While comparing PD-MCI subtypes based on the number of affected cognitive domains, the magnitude of gait impairment resulted to be directly related to the severity of cognitive dysfunction. In particular, multiple-domain PD-MCI vs. no-PD-MCI showed impaired spatial gait measures, such as shortened step and cycle length, in all three tasks, and increased measures of dynamic unbalance, similar to raised variability measures and increased double-support phase, during dual tasks. On the contrary, single-domain PD-MCI as compared to no-PD-MCI did not display any significant difference on gait features neither in single nor in dual task. Nevertheless, the *post hoc* analysis did not reveal even any significant difference when comparing gait patterns in patients with single-domain PD-MCI and patients with multiple-domain PD-MCI, thus suggesting a spectrum of dysfunction concurrently involving gait and cognition spanning across cognitively intact patients with PD, patients with single-domain PD, and patients with multiple-domain PD.

**FIGURE 1:**
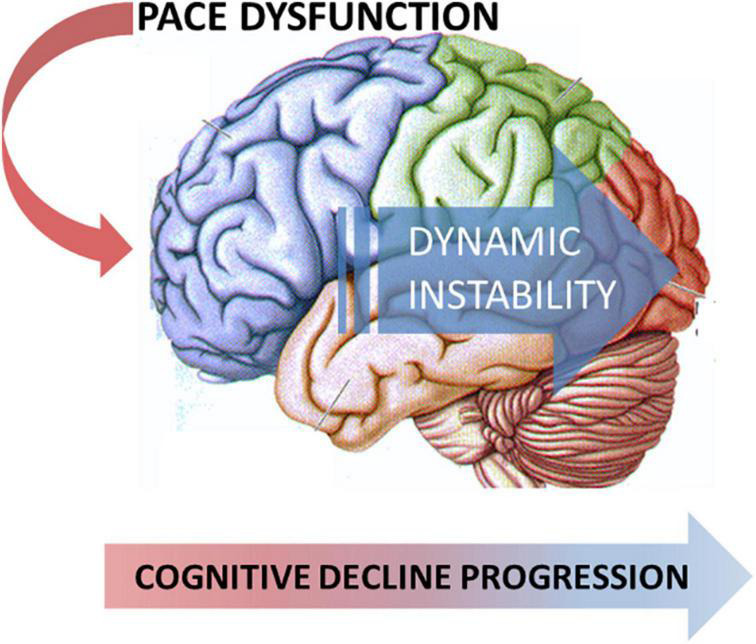
Model of the neurodegeneration progression from the anterior to the posterior cortical areas that express the concurrent occurrence of peculiar gait and neuropsychological dysfunction.

When confronting PD-MCI subtypes based on the nature of the affected cognitive domain, such as the presence of memory dysfunction, patients with amnestic PD-MCI, as compared to patients with no-PD-MCI and patients with non-amnestic PD-MCI, mainly showed increased dynamic instability during the COG task, evidenced by augmented variability measures and increased double-support phase. In addition, patients with both amnestic PD-MCI and non-amnestic PD-MCI vs. patients with no-PD-MCI subjects displayed reduced step length during the COG task. Again, we might speculate on the pairwise comparisons and infer a grading of gait dysfunction that is the greatest in patients with amnestic PD-MCI, intermediate in patients with non-amnestic PD-MCI, and the smallest in patients with no-PD-MCI. The present findings would be consistent with the progression of neurodegeneration from the anterior to the posterior cortical areas, likely involving also non-dopaminergic networks (Devos et al., 2010), which expresses with the concurrent occurrence of peculiar gait and neuropsychological dysfunction (Figure 1).

As regards walking abnormalities, previous imaging studies (Beauchet et al., 2014; Lo et al., 2017) showed that dynamic steadiness is linked to posterior brain networks, whereas more recent findings suggested the involvement of widespread cortical areas important for sensory, cognitive, and motor functions (Jayakody et al., 2020). In fact, dynamic stability is a complex activity requiring integration and control of multiple components, such as the integration of different sensory information, postural adjustments, and motor planning. As concerns cognitive dysfunction, several studies in patients with PD have demonstrated that, as the cognitive decline progresses, the cortical volume, especially in temporoparietal regions, decreases with parallel decay on memory (Lee et al., 2010; Mak et al., 2015; Pereira et al., 2015; Yildiz et al., 2015). In addition, since both multiple-domain PD-MCI and amnestic PD-MCI have been associated with the increased risk of conversion to dementia (Janvin et al., 2006; Hoogland et al., 2017; Chung et al., 2019), the increased dynamic instability, especially in dual task, might represent a possible marker of progression toward PDD. Nevertheless, further longitudinal studies are needed to investigate this speculation.

Due to the cross-sectional design of this study, these findings cannot allow any assumption about a causative relationship between posterior cortical-based cognitive deficit and dynamic instability. In other words, our findings show an association between distinctive neuropsychological profiles and specific walking abnormalities, but they cannot establish whether peculiar cognitive dysfunctions can directly contribute to the expression of particular gait patterns.

As regards the exploratory correlation analysis between spatiotemporal gait parameters during the COG dual-task and neuropsychological tests scores, only the MoCA score, a global cognition rating, correlated with gait parameters mirroring dynamic instability, thus further supporting the relationship between the severity of cognitive dysfunction and the extent of gait impairment. In fact, when analyzing correlations between domain-specific neuropsychological tests and gait variables, we found only a correlation between a visuospatial test, namely, the Benton’s Judgment of Line Orientation Test, and velocity, a raw measure underlain by multiple gait adaptations. The latter findings are not surprising since the relationship between gait and cognition would reflect common network dysfunction, expressing a collapse on both gait and cognitive skills, probably without strict correlations between walking variables and isolated cognitive test scores. Further studies on larger samples are needed to better explore this issue.

This study has some limitations. First, due to the relatively small sample size, we could not classify and, therefore, compare PD-MCI subtypes immediately according to the four categories, namely, single amnesic PD-MCI, single non-amnesic PD-MCI, multiple amnesic PD-MCI, and multiple non-amnesic PD. Although we recognized that this classification could directly show distinctive gait patterns in PD-MCI subtypes, we envisaged that the separate comparisons, i.e., single- vs. multiple-domain PD-MCI and amnestic vs. non-amnestic PD-MCI, may represent a reliable method to capture the relative burden of the quantity and quality of cognitive dysfunction on walking features. Second, since the single-domain PD-MCI group included only seven patients, this could have introduced a bias in the interpretation of data; however, this low prevalence is fairly consistent with the findings of a recent meta-analysis reporting that single-domain subtype occurs only in about one-third of patients with PD-MCI (Baiano et al., 2020). Third, we did not include an age-matched control group in our analysis. This arguably could better highlight the difference between the PD-MCI subtypes; nevertheless, a direct comparison with a control group was beyond the purpose of this study. Finally, when comparing demographic and clinical features, we found that PD-MCI vs. no-PD-MCI showed a significant difference on MDS-UPDRS III and a trend toward significance on age, and this might have had an influence on our findings. Nevertheless, such differences reflect two of the main clinical features associated with MCI, namely, older age and more severe motor symptoms, as consistently reported in the literature (Baiano et al., 2020), suggesting a tight relationship among MCI, age, and more severe motor impairment. In other terms, the gait patterns observed in MCI and its subtypes reflect the contribution of all these features that are indissociable in a real-life clinical setting.

## Conclusion

In conclusion, this study demonstrates that PD-MCI subtypes are associated with different gait patterns. Notably, both the magnitude of cognitive impairment and the presence of memory dysfunction are associated with the increased measures of dynamic unbalance, especially in dual-task condition, likely mirroring the progressive involvement of posterior cortical networks that are concurrently revealed by distinctive cognitive profiles and walking patterns. Additionally, our findings may suggest speculative clues for integrated therapeutic approaches, which could range from cognitive therapy (cognitive training interventions and use of cognitive pharmacological therapy) for improving walking performance to specific walking programs for enhancing cognitive function.

## Data Availability Statement

The raw data supporting the conclusions of this article will be made available by the authors, without undue reservation.

## Ethics Statement

The studies involving human participants were reviewed and approved by Campania Sud, the Reference Ethics Committee of the Center for Neurodegenerative Diseases of the University of Salerno. The patients/participants provided their written informed consent to participate in this study.

## Author Contributions

MA and PB contributed to conception and design of the study. CR, SC, AV, and GR organized the database. CR, LD, and MC performed the statistical analysis. MA and CR wrote the first draft of the manuscript. MP and GS wrote sections of the manuscript. All authors contributed to manuscript revision, read, and approved the submitted version.

## Conflict of Interest

The authors declare that the research was conducted in the absence of any commercial or financial relationships that could be construed as a potential conflict of interest.

## Publisher’s Note

All claims expressed in this article are solely those of the authors and do not necessarily represent those of their affiliated organizations, or those of the publisher, the editors and the reviewers. Any product that may be evaluated in this article, or claim that may be made by its manufacturer, is not guaranteed or endorsed by the publisher.

## Supplementary Material

Supplementary Figure 1 Scatterplot representing the correlation between the Benton’s Judgment of Line Orientation Test score and mean velocity under COG dual task.
