# Supplementary figures and images for: Mild Cognitive Impairment Subtypes Are Associated With Peculiar Gait Patterns in Parkinson’s Disease
Source: Front Aging Neurosci. 2022 Mar 1;14:781480. doi: 10.3389/fnagi.2022.781480 (PMC8923162; doi:10.3389/fnagi.2022.781480)

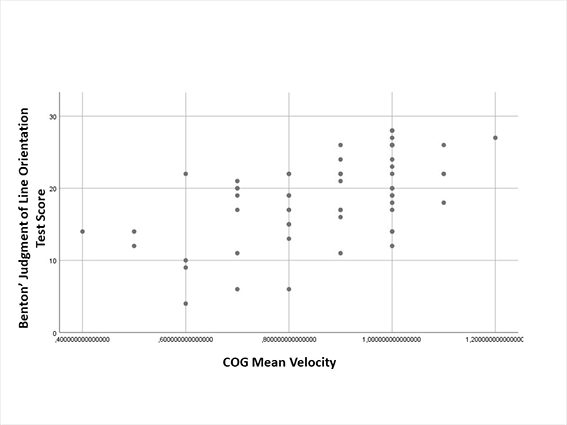

Supplement: Supplementary Figure 1 — Scatterplot representing the correlation between the Benton’s Judgment of Line Orientation Test score and mean velocity under COG dual task. [file Image_1.TIF]
